# In vivo acoustic manipulation of microparticles in zebrafish embryos

**DOI:** 10.1126/sciadv.abm2785

**Published:** 2022-03-25

**Authors:** Viktor Manuel Jooss, Jan Stephan Bolten, Jörg Huwyler, Daniel Ahmed

**Affiliations:** 1 Acoustics Robotics Systems Lab (ARSL), ETH-Zürich, Rüschlikon CH-8803, Switzerland.; 2 Department of Pharmaceutical Sciences, Division of Pharmaceutical Technology, University of Basel, Basel CH-4056, Switzerland.

## Abstract

In vivo micromanipulation using ultrasound is an exciting technology with promises for cancer research, brain research, vasculature biology, diseases, and treatment development. In the present work, we demonstrate in vivo manipulation of gas-filled microparticles using zebrafish embryos as a vertebrate model system. Micromanipulation methods often are conducted in vitro, and they do not fully reflect the complex environment associated in vivo. Four piezoelectric actuators were positioned orthogonally to each other around an off-centered fluidic channel that allowed for two-dimensional manipulation of intravenously injected microbubbles. Selective manipulation of microbubbles inside a blood vessel with micrometer precision was achieved without interfering with circulating blood cells. Last, we studied the viability of zebrafish embryos subjected to the acoustic field. This successful high-precision, in vivo acoustic manipulation of intravenously injected microbubbles offers potentially promising therapeutic options.

## Teaser

An animal-on-a-chip is designed for in vivo acoustic manipulation of microparticles in zebrafish embryos.

## INTRODUCTION

Micromanipulation methods, which include optical (*1*), optoelectronic (*2*), acoustic (*3*–*20*), or magnetic (*21*–*24*), are commonly used to elucidate biological processes at the cellular level. However, studies involving micromanipulation are often conducted in vitro, which does not fully reflect the complexity of in vivo cellular environments. Successful transition of micromanipulation methods to in vivo use will have particularly substantial impacts in the fields of cancer research, brain research, and vasculature biology.

Recently, researchers have used optical tweezers to trap and manipulate particles and cells in small animal models, for example, within the vasculatures of zebrafish embryos (ZFEs) (*25*) and in the almost-transparent blood vessels in the ears of mice (*26*). In addition, optical tweezers have been used to manipulate otoliths to control vestibular behaviors in larval zebrafish (*27*, *28*). Notably, although the high spatial resolution of optical tweezers makes them attractive for use in small model organisms, optical methods are restricted to transparent samples. Other drawbacks of optical methods are their association with bulky, complex, and expensive setups that may be difficult and expensive to miniaturize and their high power. Optical methods can cause physiological damage due to laser-induced heating and multiphoton absorption in biological materials (*29*). It is also worth noting that optical tweezers have limited penetration depth, on the scale of millimeters, which is not sufficient for manipulation tasks in larger animals.

Magnetic systems have the potential to open up manipulation to nontransparent samples and larger penetration depths. For example, injection of magnetic microparticles into zebrafish and mouse embryos has been used to enable three-dimensional (3D) rotational manipulation for microscopy (*30*). Magnetically responsive ferrofluid microdroplets have been used to quantify anisotropic stress in a mouse embryo (*31*). Magnetic micro- and nanoparticles have also been used for in vivo drug delivery in mice, and more recently, magnetic microrobots and swarms have been successfully navigated inside mice (*32*). However, magnetism-based methods have the fundamental drawback of depending on particles that have been doped with magnetic materials. In addition, when in the animal body, the particles are cleared through iron metabolism, which can take months from the time of administration (*33*, *34*).

Acoustic methods for in vivo micromanipulation represent an exciting technology that has potential to bypass the abovementioned problems. In general, the radiation forces associated with acoustic systems are large, typically in the micro- to nanonewtons (*35*). A recent study demonstrated the use of an ultrasound beam consisting of 256 piezo elements to trap a 3-mm glass bead inside the urinary bladder of a pig (*36*). Another used four piezo elements to develop an acoustic vortex generator and manipulate microparticles within rodent vasculatures (*37*). In the context of small model organisms such as *Caenorhabditis elegans* and zebrafish, acoustic manipulation technology has been widely used for organism-level manipulation and levitation, but no work to date has been conducted on controlling foreign particles inside small-organism vasculatures (*3*, *38*–*41*). In vivo manipulation inside a zebrafish requires sufficient force to overcome drag, selective trapping of a microparticle of interest, and high-precision manipulation on account of vasculature micrometer dimensions. Here, we report the development of an in vivo acoustic manipulation method that addresses these fundamental challenges. Manipulation of injected microparticles to a spatially targeted location in ZFEs could open up new approaches for targeted drug therapies and tightly controlled toxicity studies.

Zebrafish are small aquatic vertebrates widely used as a standard animal model in research on account of their small size, transparent bodies, rapid embryonic development process, and relatively simple genetic manipulation. Numerous genetic zebrafish disease models have been used to study clinically relevant disorders including heart disease, anemias, cancer, and diseases of the nervous system (*42*). The ZFE model thus serves as an alternative, high-throughput, cost-effective method for drug screening and is frequently used to identify cures. However, current drug screening methodologies cannot avoid exposing the entire fish to the drug of interest. More accurate effects and conclusions could be realized by specifically delivering a drug to the tissue or site where its effect is desired; for example, delivering a heart disease medication directly to the heart would enable precise observation of its metabolism, toxicologic effects, and elimination in that tissue. Furthermore, selective delivery by means of micromanipulation can aid in decreasing off-target effects and increasing therapeutic efficacy.

Here, we present an in vivo acoustic manipulation device and demonstrate microparticle manipulation under blood flow in the vasculatures of ZFEs. In particular, we show acoustic manipulation of gas-filled microbubbles with micrometer precision along with 2D spatial control of a microbubble. We achieved this by designing the zebrafish chamber to be off-center and applying frequency modulation control of the acoustic field. We also demonstrate reversible manipulation, i.e., controlled back and forth motion, of microbubbles in the intersegmental vessels (ISVs) and the cerebral blood vessels of ZFEs. Last, we confirmed the viability of ZFEs in the presence of the acoustic field.

## RESULTS

### Experimental setup

**Fig. 1.**
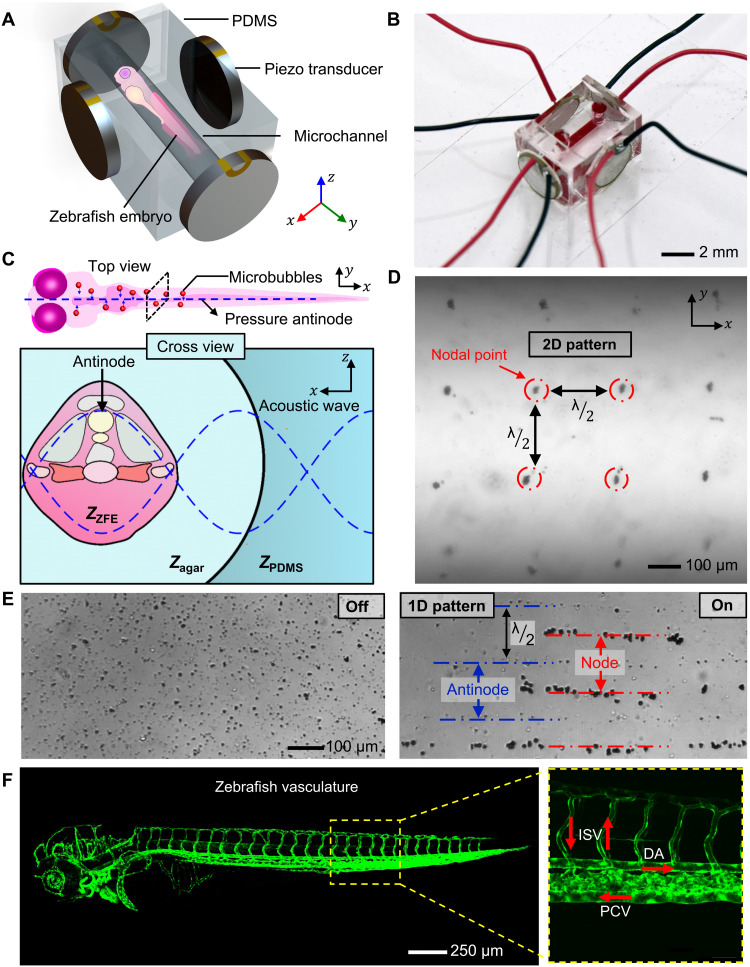
Experimental design and concept of the in vivo acoustic manipulation system. (**A**) Schematic of the in vivo acoustofluidic device, which comprises a transparent PDMS chamber for the ZFE and four identical piezo transducers. (**B**) Micrograph of the in vivo acoustic manipulation chamber. Red dye was infused to highlight the channel. (**C**) Schematic of an acoustic wave penetrating a ZFE. A standing acoustic wave is established across a ZFE that is fixed in agar gel. When the acoustic wave encounters the PDMS/agar and agar/ZFE interfaces, its energy is partially reflected and partially transmitted (see also table S1). (**D**) The 2D lattice-like acoustic pattern is produced inside the microchannel by the acoustic system. (**E**) The 1D patterning demonstrates microbubbles that have resonance frequencies larger than the excitation frequency positioned at the pressure antinodes, while microbubbles having smaller resonance frequency than the excitation frequency are trapped at the nodes. An ultrasound induced 1D patterning of gas-filled microbubbles. The images illustrate the distribution of microbubbles before and after applying ultrasound. In the 1D pattern, microbubbles with resonance frequencies higher than the excitation frequency are shown positioned at the pressure antinodes, while microbubbles with resonance frequencies lower than the excitation frequency are seen trapped at the nodes. (**F**) Fluorescence image of a tg(kdrl:eGFP) ZFE, expressing green fluorescent protein (GFP) in endothelial cells, highlighting its vasculature network. Red arrows indicate direction of blood flow, e.g., dorsal artery (DA), intersegmental vessels (ISV), and posterior cardinal vein (PCV).

Our in vivo acoustic manipulation device was composed of four identical piezoelectric actuators and a polydimethylsiloxane (PDMS)–based transparent polymer chamber accommodating a circular channel in which the ZFE was arranged (see also fig. S1). The piezoelectric actuators, with thickness-mode resonance frequency of 4.25 MHz, were positioned orthogonally to each other; see Fig. 1A. The zebrafish sample holder was positioned 2 mm off-center with respect to the orthogonally positioned transducer pairs, thus enabling a wider manipulation range of ~100 μm. PDMS was selected as the material for the acoustic manipulation chamber due to being optically and acoustically transparent and chemically inert (*43*). Since the ZFE is also optically transparent, the combination with PDMS is ideally suited for use on inverted microscopes. For the experiment, clinically used ultrasound imaging agents “SonoVue” were carefully injected into the vasculature of ZFE at 72 hours postfertilization (hpf) by a high-precision pneumatic pico-pump. The microbubbles contained sulfur hexafluoride enclosed by a thin lipid-monolayer membrane, thus extending the stability of the microbubbles up to 6 hours (*44*, *45*). The embryo was carefully positioned in the circular channel and subsequently immobilized using an agar gel to ensure that it could not swim away or be made to drift by the presence of an acoustic field (see Materials and Methods). A pair of electronic function generators connected to the piezoelectric transducers were used to maneuver the microbubble in vivo. The excitation ultrasound frequencies were modulated from 4.0 to 4.25 MHz, and an applied peak-to-peak voltage (*V*_PP_) between 1 and 17.5 *V*_PP_ was used. The whole setup was positioned on an inverted microscope with phase imaging capabilities to visualize and track the microbubbles inside the vasculature. Last, experimental images and videos were captured using high-sensitivity and high-speed cameras.

Here, we have developed an in vivo acoustic manipulation system using clinically approved, commercially available, biocompatible gas-filled polymeric-shelled microbubbles. The intravenously injected microbubbles circulated freely within the vasculature of the ZFE. They drifted through the heart down the dorsal aorta (DA) toward the tail end of the embryo and then recirculated back into the heart through the posterior cardinal vein (PCV).

When acoustic waves propagate through ZFE sample holder, the incident acoustic energy is transmitted and reflected first at the PDMS/agar and then at the agar/ZFE interface according to the acoustic impedance mismatches (see also Fig. 1C and fig. S2). The acoustic impedance, *Z*, of a material can be computed as *Z* = ρc, where ρ and c are the density of the material and the speed of sound in that material, respectively. We assume that the embryo has properties similar to that of other soft biological samples as listed in table S1 (*43*, *46*, *47*). The transmission is expressed by the intensity transmission coefficient, *T*. At the interface between the embryo and the agar gel, the transmission can be calculated as $T=1-{\left[\frac{Z_{\text{ZFE}}-Z_{\text{agar}}}{Z_{\text{ZFE}}+Z_{\text{agar}}}\right]}^{2}$, where *Z*_agar_ is the impedance of the agar solution, while *Z*_ZFE_ is the impedance of the ZFE. Since the acoustic impedance of PDMS, agar, and ZFEs is similar to each other (see also table S1), the acoustic wave intensity is transmitted 96.1% at the PDMS/agar interface and 99.9% at the agar/ZFE interface. The transmission through the soft materials is more than 90%. Thus, the size or geometry of ZFEs should not affect acoustic trapping.

As a pair of oppositely positioned horizontal piezoelectric transducers are activated at a similar frequency and amplitude, a 1D standing acoustic wavefield develops (*48*). The wavefield comprises of a series of pressure nodal and antinodal lines, which are separated by quarter wavelengths (see also Fig. 1E). This distance $d=\frac{c}{4f}$ depends on the speed of sound of the material and actuation frequency. Similarly, if a second set of piezo transducers in the *x* direction is actuated, then they produce a standing wavefield propagating in the *x* direction. The two sets of wavefields interfere, and their superposition produces a 2D standing acoustic wavefield with nodal and antinodal points (*48*). The distance between each nodal point was measured to be 182 ± 17 μm at 4.2 MHz when actuated with 5 *V*_PP_. The 1D and 2D lattice-like acoustic pattern produced inside a circular channel is shown in Fig. 1 (D and E) (see also movies S1 and S2).

When acoustic waves travel through a liquid containing microparticles, they induce a time-average acoustic radiation force on the particles (*49*). This force, which results from the scattering of incident waves, is composed of primary and secondary radiation forces. The primary radiation force arises due to interactions of particles with the standing wavefield. The primary radiation force, $F_{R}(x)=4\pi \phi (\tilde{\kappa },\tilde{\rho })k_{x}a^{3}E_{a}\text{sin}(2k_{x}x)$, on a small spherical compressible particle at position *x* in an acoustic pressure field can be estimated from the time-averaged gradient of the Gor’kov potential (*49*, *50*). The radius *a* of the particle in this case is much smaller than the acoustic wavelength λ in a 1D standing wavefield of wave number *k_x_*. *E*_a_ denotes the acoustic energy density. The acoustophoretic contrast factor $\Phi =\frac{1}{3}[\frac{5\tilde{\rho }-2}{2\tilde{\rho }+1}-\tilde{\kappa }]$ determines the directionality of the radiation force; $\tilde{\rho \;}=\frac{\rho_{s}}{\rho_{0}}$, where ρ_0_ and ρ_s_ respectively denote the density of the liquid and of the particle; $\tilde{\kappa }=\frac{\kappa_{s}}{\kappa_{0}}$, where κ_0_ and κ_s_ respectively denote the compressibility of the liquid and the particle. The acoustophoretic contrast factor Φ for polystyrene particles is +0.24 in water, suggesting that they will move to the nodal pressure lines. In contrast, the behavior of the shelled microbubbles in an acoustic field is size dependent. Depending on size, the resonance frequency ranges between 1 and 9 MHz, as indicated by the manufacturer (*44*, *51*, *52*). Microbubbles that have resonance frequencies larger than the excitation frequency move to the pressure antinodes, while those having a resonance frequency smaller than the excitation frequency move to the pressure nodes (*51*, *52*). To clarify the trapping locations of the microbubbles, we filled a microchannel with SonoVue microbubbles. Under excitation frequencies between 4.0 and 4.25 MHz, bubbles smaller than 4 μm move to the antinode, while those larger than 4 μm move to the node (shown in Fig. 1E). A wide range of microbubbles and clusters of microbubbles (~6 to 14 μm) were detected in the in vitro experiments. The microbubbles used in the ZFE therefore move toward the pressure node (see also fig. S3 and movie S2).

### In vivo microbubble trapping

**Fig. 2.**
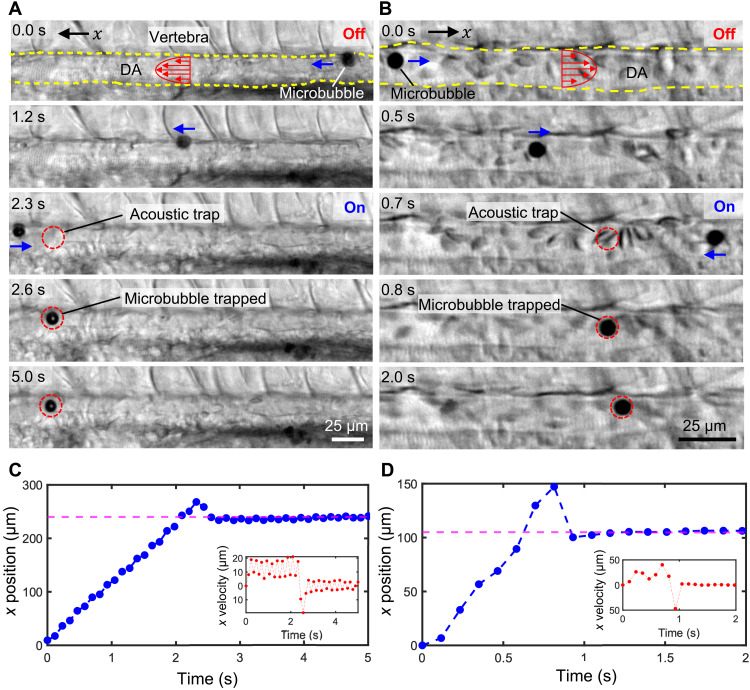
Microbubble manipulation inside the DA of ZFEs. (**A**) Image sequences demonstrating a gas-filled microbubble migrating under right-to-left flow that became trapped in the DA when ultrasound was activated (at ~2.3 s). The microbubble traveled against flow to reach the antinode, indicated by the dotted red circle. (**B**) A microbubble traveling under left-to-right flow in the DA of another fish similarly became trapped. Note that the microbubbles are traveling much faster upstream than downstream, which can be attributed to acoustic radiation force. The ZFE was observed to drift vertically 7.6 μm (smaller than the wavelength of 365 μm). (**C**) The plots illustrating the location and speed (inset) of a microbubble tracked for the image sequence in (A). A magenta dotted line indicates the location of the pressure node. (**D**) Plots of the *x* position and the *x* velocity (inset) of the microbubble in (B). The pressure nodal position is indicated by magenta dotted lines.

In this section, we focus on trapping a freely recirculating gas-filled microbubble in the vasculature of a ZFE. In Fig. 2 (A and C), a microbubble circulates from right to left in the DA until the acoustic field is turned on at 2.3 s. The consequent primary acoustic radiation force, *F*_R_, moves the bubble into the nearest acoustic trap (node), which is located to the right, against the flow (see also movie S5). Figure 2 (B and D) depicts an equivalent experiment in which the head of the fish is oriented in the opposite direction; thus, the flow direction is from left to right. As in the previous experiment, a microbubble inside the DA drifts downstream until the acoustic field is turned on at 0.7 s, upon which the microbubble travels toward the nearest acoustic trap, in this case, upstream to the left (movie S6). A ZFE fixed in agar may occasionally drift slightly when subjected to ultrasound over a period of a few seconds.

In addition to the primary acoustic radiation force, *F*_R_, the bubble experiences “Stokes” drag force, *F*_D_ (*49*). The influence of inertia forces in this experiment is characterized by the Reynolds number. The Reynolds number, $\text{Re}=\frac{\rho a\text{∆}v\;}{\mu }$, of a microbubble circulating inside the vasculature is measured to be 3.23 × 10^−6^, where ρ = 1010 kg m^−3^ is the density of human blood plasma, *a* = 6 μm is the diameter of the microbubble, μ = 1.5 cP is the dynamic viscosity of the blood plasma, and ∆*v* ~ 0.8 μm s^−1^ is the velocity difference between bubble and medium (*53*, *54*). There is no literature on the density of ZFE blood plasma. The physical properties of human blood and ZFE blood are similar (*55*). We therefore approximate the ZFE blood plasma density as the one of human blood plasma. The velocity of the bubble’s transit upstream is determined empirically from an image sequence, and the velocity of the blood flow is approximated as the drifting speed of the bubble in the absence of acoustic force. Since the calculated Reynolds number is low (Re ≪ 1), inertia effects become negligible; thus, the acoustic radiation force is balanced by the Stokes drag, i.e., *F*_R_ = *F*_D_. Therefore, we can estimate the maximum acoustic radiation force acting on the microbubble as *F*_R_ = *F*_D_ = 6πη*a*∆*v*, which is in the order of piconewtons (see also Supplementary Text).

In contrast to microbubbles, we observed that the red blood cells (RBCs) in the vasculature were hardly affected in the presence of ultrasound. Microbubbles and cluster of microbubbles detected in the ZFE had a diameter of ~6 μm, while the observed RBCs had a diameter of ~6 μm. The Stokes drag acting onto the microbubbles is therefore similar in magnitude compared to the RBCs. Microbubbles (0.004 × 10^6^ $\frac{\text{kg}}{m^{2}\;s}$) have a larger acoustic impedance mismatch with the surrounding blood plasma (1.57 × 10^6^ $\frac{\text{kg}}{m^{2}\;s}$) than RBCs (1.6 × 10^6^ $\frac{\text{kg}}{m^{2}\;s}$) (*41*). The acoustic pressure wave scatters more at the microbubbles, which results in a higher acoustic radiation force acting on the microbubbles. As a result of much larger acoustic radiation force, the microbubbles are selectively manipulated by the acoustic field.

### In vivo microbubble manipulation

Next, we demonstrate controlled dynamic manipulation of trapped microbubbles inside a ZFE. Trapped microbubbles can be made to translate by changing the nodal positions of a standing wavefield, i.e., by tuning the excitation frequency emitted by the piezo transducer. A change in frequency Δ*f* results in a change in wavelength Δλ. Since the microbubbles are trapped at the nodes, they move with the node shift; however, the effective range of this manipulation is extremely small (see also movie S3). The piezoelectric transducers mounted to the setup have a quality factor *Q* of 26 to 93 and achieve maximum oscillation when activated at their resonance frequency of 4.05 to 4.17 MHz (see also figs. S6 and S7). Driving the transducers off-resonance decreases the mechanical output amplitude, thus limiting effective translation of the microbubbles. Within the frequency window of 4.0 to 4.25 MHz, the difference in wavelength in water is approximately ∆λ = λ_1_ − λ_2_ ≈ 23 μm.

**Fig. 3.**
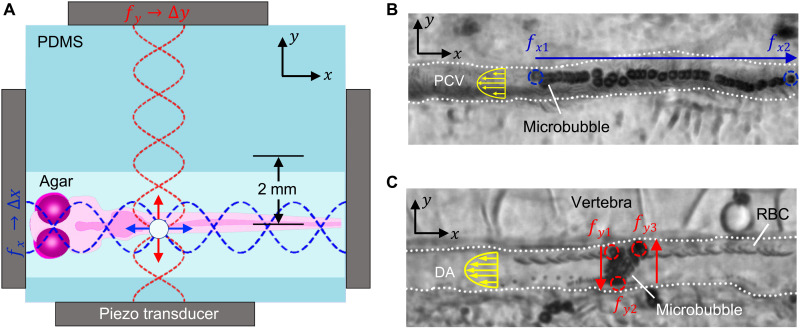
2D control of a microbubble using frequency modulation control. (**A**) A schematic demonstrates the zebrafish chamber positioned 2 mm off-center with respect to the orthogonally positioned transducer pairs enabling a wider manipulation range. (**B**) A microbubble is made to move in the *x* direction against the flow as the transducers placed in line with the fish have their frequencies changed from *f*_*x*1_ = 4.0 MHz to *f*_*x*2_ = 4.25 MHz, 15 *V*_PP_, while *f_y_* = 4.1 MHz is kept constant at 12.5 *V*_PP_. (**C**) Equivalently, altering the excitation frequency of the piezo transducers alongside the ZFE from *f*_*y*1_ = 4.0 MHz to *f*_*y*2_ = 4.25 MHz at 12.5 *V*_PP_ and back to *f*_*y*3_ = 4.1 MHz at 12.5 *V*_PP_ thus results in movement of the microbubble in the *y* direction, i.e., perpendicular to the blood flow. In the process, the particle remains stable in the *x* direction as *f_x_* = 4.1 MHz is kept constant.

To increase the range of motion, we positioned the fish chamber 2 mm off-center relative to the wavefield, shown schematically in Fig. 3A. In a standing wavefield, each node location is sited half a wavelength (λ/2) away from its adjacent antinodes. A change in frequency Δ*f* results in a change in wavelength Δλ; the node positions therefore shift when the excitation frequency is modulated, with only the node at the center of the wavefield remaining stationary. The displacement of each node increases proportionally with its distance from the center (*3*); that is, when we change the wavelength by Δλ, the innermost antinodes, which are half a wavelength (λ/2) from the central node, will move by Δλ/2 ; the next tier out, which is one wavelength (λ) from the center, moves by Δλ; the ones that are two wavelengths (2λ) from the center move by 2Δλ; and so forth. Siting the chamber five wavelengths (5λ = 2 mm) off-center made the node translations five times larger than the shift in wavelength (Δλ = 23 μm), producing a net translation of 5 × 23 = 115 μm (see also figs. S6 and S7 and movie S4).

The piezo transducers arranged to the left and right of the ZFE control the *x* position of the microbubble, while those above and below control its *y* position (Fig. 3A). Figure 3B shows a superimposed time-lapse image of a microbubble that was manipulated right to left by increasing the frequency of the *x*-axis piezo transducers from 4.0 to 4.25 MHz at 15 *V*_PP_, while the *y*-axis piezo is kept constant at 4.1 MHz and 12.5 *V*_PP_. The microbubble traveled 106 μm upstream in the PCV, i.e., against blood flow, over the course of 13 s (movie S7). This movement of 106 μm is 92% of the maximal net translation of the device. Likewise, Fig. 3C illustrates a microbubble maneuver in the *y* direction within the 16-μm-wide DA as the *y*-axis piezo transducers were switched from 4.0 to 4.25 and back to 4.0 MHz at 12.5 *V*_PP_, causing the microbubble to move orthogonally to the flow (movie S9). During this process, the *x*-oriented transducers were maintained constant at a frequency of 4.1 MHz and amplitude of 12.5 *V*_PP_ sufficient to counter the blood flow, thus holding the microbubble in the *x* direction. The microbubble does not move further than 16 μm, as it is blocked by the vessel walls of the DA. Such complete 2D control of a microrobot inside a vasculature under flow would allow the navigation of injected carriers through the body to a target location. Therefore, this approach could provide a foundation for future locally targeted drug delivery or spatially selective drug screening.

### Manipulation of microbubbles in different regions

**Fig. 4.**
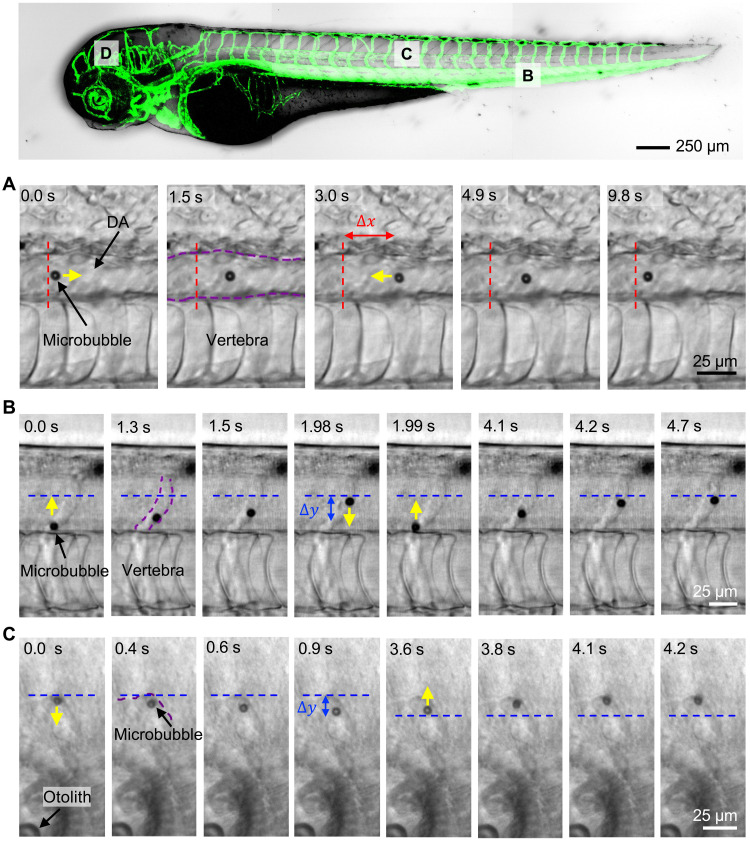
Acoustic manipulation in vivo at different locations within the zebrafish vasculature. To demonstrate full directional control, a bubble was directed along the vasculature and back to its initial position multiple times by shifting the frequency. (**A**) The microbubble was reversibly moved horizontally 22 μm left and right in the 21-μm-wide DA by varying the frequency controlling the horizontal position *f_x_* between 4.1 and 4.15 MHz at a constant amplitude of 10 *V*_PP_. (**B**) The bubble likewise traveled and returned 29.85 μm in the *y* direction without stiction in the ISV, which has a diameter close to that of contrast agents and passing RBCs by varying the frequency controlling the vertical position *f_y_* between 4.15 and 4.25 MHz at a constant amplitude of 12.5 *V*_PP_. (**C**) Last, the same reversible behavior was demonstrated with a traveled distance of 9 μm in a cerebral blood vessel, close to the otoliths by varying the frequency controlling the vertical position *f_y_* between 4.1 and 4.25 MHz at a constant amplitude of 10 *V*_PP._

We demonstrated manipulation of microbubbles in the DA and the PCV, which are the two largest vessels in the ZFE exhibiting the highest flow rates (*56*). Subsequent experiments investigate whether this acoustic-directed movement is repeatable and reversible throughout the body in different regions. With this control, a microbubble could be injected near the heart and manipulated to an arbitrary location in the vasculature. In Fig. 4A, a constant frequency was applied in the *y* direction such that the bubble was fixed in the middle of the DA. Meanwhile, switching the frequency between 4.1 and 4.15 MHz at a constant amplitude of 12.5 *V*_PP_ of the *x*-direction actuators manipulated the bubble upstream by 26 μm. Afterward, reversal of that actuation frequency caused the bubble to travel back to its initial position. The final location of the bubble deviated from its initial position by 5 μm (movie S11). Relative to the DA, other segments of the vasculature with smaller diameters experience lower flow rates. Figure 4B shows repeated up and down movement inside the ISV over a distance of 21 μm. This motion is caused by the actuators located above and below the ZFE with varying frequency between 4.15 and 4.25 MHz at a constant amplitude of 12.5 *V*_PP_ (movie S12). Last, we demonstrated tweezing of microbubbles at sites other than in the fish’s tail, specifically inside the cerebral vasculature (Fig. 4C). In this case, the bubble was moved away from the vasculature wall by 9 μm and then back to its original position at the wall by varying the frequency controlling the vertical between 4.1 and 4.25 MHz at a constant amplitude of 10 *V*_PP_ (movie S13). This reversible back and forth movement throughout the body supports this system as a possible conceptual foundation for localized applications.

**Fig. 5.**
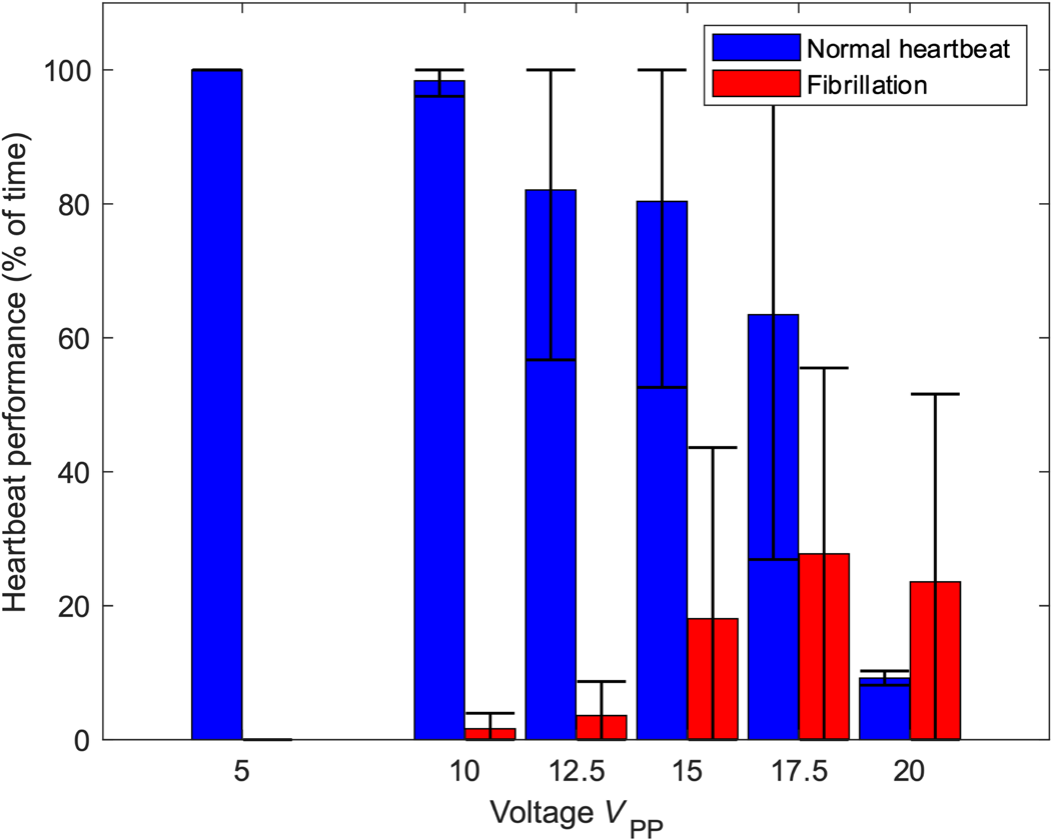
Viability of ZFE in the presence of ultrasound. To demonstrate the viability of the acoustic actuation on cardiac function, untreated ZFEs were placed into the acoustic channel and exposed to acoustic excitation across a range of voltages in pulses of 30 s with a subsequent recovery time of 1 min. Up to a voltage of 17.5 *V*_PP_, fibrillation was reversible and did not impair ZFE viability. The error bar represents the SD for a minimum of five data points.

### Viability study of ZFEs

A ZFE at 72 hpf has a total length of ~3.5 mm, and its whole body experiences acoustic radiation force. We expect that imposing this force on tissue and RBCs puts stress on the cardiovascular system. The ZFEs in our experiments experience an acoustic field in pulses of 30 s with a subsequent recovery time of 1 min. We observe a voltage-dependent reaction in heart rate and pumping at voltages above 10 *V*_PP_ (see Fig. 5). The acoustic radiation force scales with the square of the actuation voltage. We observed that voltages between 12.5 and 15 *V*_PP_ applied to the piezo transducer can lead to fibrillation (temporary erratic behavior of the heart) and can stop the heart for a limited amount of time (~30 s); however, after the application of acoustics has ceased, the heart recovers back to its initial movement within 1 min (see also movies S14 and S15). In contrast, a voltage of 17.5 *V*_PP_ stops the heartbeat within 30 s. After ceasing such excitation, the stopped heart in some specimens starts fibrillating within 30 s and may fully recover in minutes. At a still-higher excitation voltage of 20 *V*_PP_, permanent deformation can result, i.e., buckling along the long axis of the zebrafish body. We therefore conclude that an excitation voltage of no more than 12.5 *V*_PP_ or limited excitation time at values of up to 17.5 *V*_PP_ will ensure the continuing viability of a ZFE. Last, we investigated whether the electric field produced by the piezo transducers has any effect on the cardiac function of the ZFE. A similar viability study was conducted with an acoustically isolated 1-mm air gap between the transducer and the PDMS chamber, and no changes in heart rate were detected (see also movie S16). For higher animal models, the standing wavefield is smaller than the body size; thus, the acoustic field does not completely excite the animal’s entire body. The cardiovascular effect of the acoustic field in mice or humans is therefore presumably less intense, increasing the viability of acoustic manipulation.

## DISCUSSION

Here, we demonstrate microparticle manipulation inside ZFEs using ultrasound. Ultrasound is attractive for its ability to penetrate deep into tissue, for being unaffected by tissue opacity, and for generating a broad range of directed forces. We used ultrasound to manipulate microbubbles with micrometer precision inside the DA of zebrafish. The setup demonstrates controlled up-, down-, and cross-stream manipulation throughout the vasculature by controlling the acoustic actuation frequency. We further realized controlled manipulation of microbubbles in ISVs and cerebral blood vessels. Last, we investigated the viability of ZFEs subjected to the acoustic field.

After injection the microbubbles circulate freely inside the fish for up to 15 min in the absence of any acoustic fields. As an acoustic field excites the bubbles, they clump together into groups due to secondary “Bjerknes” forces (*57*) as they move into an acoustic trap. The enlarged cluster of bubbles can reduce circulation time in the fish depending on the number of injected bubbles. The bubbles remain in clusters after the acoustic field has been turned off for around 30 s. However, stiction of single or groups of bubbles inside the zebrafish vasculature has not been observed to be fatal. A control over the number of injected bubbles may result in less agglomeration of bubbles throughout the vasculature.

Our system shows reliable trapping of bubbles and patterning of particles in water of a 1.5-mm-wide channel. A subsequent change in actuation frequency moves the position of the trap and thereby the trapped bubble. During in vivo experiments, the acoustic node may be located within the ZFE tissue but outside the 5- to 25-μm-wide vasculature walls. As a result, the position of trapped injected microbubbles may differ from the actual node location. Without the visual feedback of the precise node position, the execution of a desired frequency shift to precisely move a trapped microbubble is challenging. Furthermore, we observed a deviation between the output signal of the piezo transducer and the excitation signal applied to the function generator at the resonance frequency of the transducers (see also fig. S4). A more sophisticated frequency control with, for example, a displacement transfer function or autocalibration might simplify path planning of bubbles inside the fish.

As acoustic waves penetrate different biological tissue, they get scattered, transmitted, and attenuated. We have not observed bulk acoustic streaming in the zebrafish vasculature under the experimental conditions used in this study. Therefore, bulk streaming in the form of “Rayleigh” or “Eckart” streaming should not compromise the performance of acoustic trapping. Our results show that transmission is sufficiently large to acoustically manipulate particles under flow in ZFE throughout the whole body of the ZFE, independent of tissue type, flow conditions, vessel diameter, or incident angle. Larger animal models have a longer distance between actuator and circulating microbubble. A resulting increased attenuation might limit location-dependent acoustic in vivo manipulation.

Microbubbles in combination with ultrasound have already been shown to trigger drug release (*58*). Here, we now present controlled in vivo manipulation of microbubbles in the vasculature of a ZFE. We believe that spatial control of drug-loaded microparticles can be exploited for targeted drug delivery. Additional studies are needed to demonstrate that the observed effects in ZFE can be extrapolated to higher vertebrate including humans.

## MATERIALS AND METHODS

### Experimental setup

The acoustic chamber into which a microbubble-injected ZFE was embedded was transferred to a Zeiss AxioVert 200M (Carl Zeiss, Germany) inverted optical microscope equipped with 2.5×, 5×, and 10× objectives. During experiments, the voltage signals driving the piezo actuators were produced by a Tektronix AFG 3011C function generator (Tektronix, USA) and a GW Instek AFG-2005 function generator (GW Instek, Taiwan). The conductance and impedance of four nonmounted and four mounted piezoelectric transducers were measured using an impedance analyzer (16777K, SinePhase, USA) (see also fig. S5 and table S2). Input power was assessed by measuring the voltage across the transducer and current at the transducer. A “TBS2000” oscilloscope by “Tektronix” was used to measure the voltage across the piezo transducer and a “TCP202” current probe by Tektronix to measure the current to the piezo transducer. By multiplying the root mean square (RMS) voltage and RMS current at the piezo, we computed the apparent input power to the piezo. Images were taken with a Coolsnap HQ2 charge-coupled device camera (Photometrics, USA) at a frame rate of 8.6 fps and processed with PVCamTest software (Photometrics, USA). Subsequent image analysis was performed with FIJI, a package based on ImageJ (imagej.net, USA). For the visualization of the ZFE vasculature in Fig. 1, confocal images of tg(kdrl:eGFP) embryos were acquired using an Olympus FV3000 confocal laser scanning microscope (Olympus, Tokyo, Japan) equipped with a UPIabSApo 20× objective having a numerical aperture of 0.75 (Olympus, Japan). Image acquisition was carried out using a sequential line scan, an excitation wavelength of 488 nm, and an emission wavelength of 500 to 540 nm.

### ZFE husbandry

In accordance with the Swiss animal welfare regulations, eggs from *Danio rerio* AB/TU wild-type and kdrl:eGFP [green fluorescent protein (GFP) expressed by endothelial cells] were maintained. They were kept in zebrafish culture media at 28°C, to which 1-phenyl 2-thiourea (30 μg/ml; PTU) was added to prevent the formation of pigment cells. At 3 days after fertilization, ZFEs were anesthetized with 0.01% tricaine, dechorionized, and embedded in 0.3% agarose containing tricaine and PTU.

### ZFE injection

A volume of 2 to 3 nl of SonoVue microbubble solution (Bracco, Italy) was injected into the duct of Cuvier of embedded ZFEs using a micromanipulator (Wagner Instrumentenbau, Schöffengrund, Germany), a pneumatic Pico Pump PV830 (World Precision Instruments, USA), and a Leica SAPO microscope (Leica, Germany). After injection, the ZFEs were manually cut out of the agar, transferred into the acoustic chamber, and re-embedded using low-melting agar.
