# Supplementary material for: In vivo acoustic manipulation of microparticles in zebrafish embryos
Source: Sci Adv. 2022 Mar 25;8(12):eabm2785. doi: 10.1126/sciadv.abm2785 (PMC8956268; doi:10.1126/sciadv.abm2785)
Supplement: Supplementary file 1 — Supplementary Text Figs. S1 to S7 Tables S1 and S2 [file sciadv.abm2785_sm.pdf]

Science Advances
AAAS

Supplementary Materials for

In vivo acoustic manipulation of microparticles in zebrafish embryos

Viktor Manuel Jooss, Jan Stephan Bolten, Jörg Huwyler, Daniel Ahmed*

*Corresponding author. Email: dahmed@ethz.ch

Published 25 March 2022, *Sci. Adv.* **8**, eabm2785 (2022)
DOI: 10.1126/sciadv.abm2785

The PDF file includes:

Supplementary Text
Figs. S1 to S7
Tables S1 and S2
Legends for movies S1 to S16

Other Supplementary Material for this manuscript includes the following:

Movies S1 to S16

Supplementary Text

Resonance of experimental setup

The piezo actuators used in the experiments operate at their first thickness mode resonance. Depending on the quality factor (Q) of the piezo crystal and the experimental setup the deformation amplitude increases around the resonance frequency (f_{res}). A frequency sweep measurement of the piezos in fig. S5 characterizes conductance and impedance. The resonance frequency is determined from the minima of the real part of the electrical impedance. The bandwidth is measured from the frequency window at which the amplitude reaches 70.7% of the resonance amplitude. The Q factor can be calculated as the ratio between resonance frequency and bandwidth. The measured resonance frequency of the unmounted transducers varies between 4.140 MHz to 4.134 MHz, which is lower compared to the resonance frequency of 4.25 MHz indicated by the supplier. The corresponding quality factor of the unmounted transducers ranged from 225.6 to 320.5. When the transducers were glued onto an acoustofluidic setup, that was filled with water, the resonance frequency decreases slightly into the range of $4.048 - 4.173$ MHz. Since additional losses and attenuation are introduced when the piezo actuators are mounted onto the setup the quality factor decreases to $26.3 - 93.0$. The impedance measurements agree reasonably well with the operational frequency window during the experiments of $4.0 - 4.25$ MHz.

Fabrication

The PDMS acoustofluidic chamber shown in fig. S1C is fabricated from three different molds, one for the channel and two endcaps. The first mold shown in fig. S1A consists of a glass capillary with an outer diameter of 1.55 mm, glass slides, and 3D printed parts that hold the components together. The two other molds shown in fig. S1B are each made up of two glass slides and a 3D printed frame. A SLA 3D printer (Form 3 by Formlabs, Germany) was used to fabricate the non-glass parts of the molds. Before every use, the molds were silane-coated in a vacuum chamber for two hours to improve the release of the cured parts. To construct the acoustic chamber, uncured Sylgard 184 Silicone Elastomer Base and Sylgard 184 Silicone Elastomer Curing Agent (Dow Corning, USA) were first mixed at a 10:1 ratio (weight: weight) for five minutes. The resulting PDMS was placed in a vacuum chamber for 30 minutes to remove air bubbles, then poured into the molds depicted in fig. S1A-B and cured in an oven at 85 °C for four hours. Subsequently, the cured PDMS blocks were removed from the molds. A 3D printed template was used in shortening the PDMS blocks to the desired size, after which a PDMS puncher was used to create a 1.5 mm wide inlet and outlet above the microchannel. The two endpieces were electro-bonded onto the center block containing the microchannel, after which the whole construct was electro-bonded onto a glass slide shown in fig. S1C. Four piezo discs (7 x 0.5 mm R Wire Leads, Steminc, USA) with a resonance frequency of 4.25 MHz were glued onto the sides of the acoustic chamber with UHU Epoxy Plus Sofortfest (UHU, Germany) and left to cure for 20 minutes. A 3D printed template ensured accurate positioning of the piezo discs.

Supplementary Figures

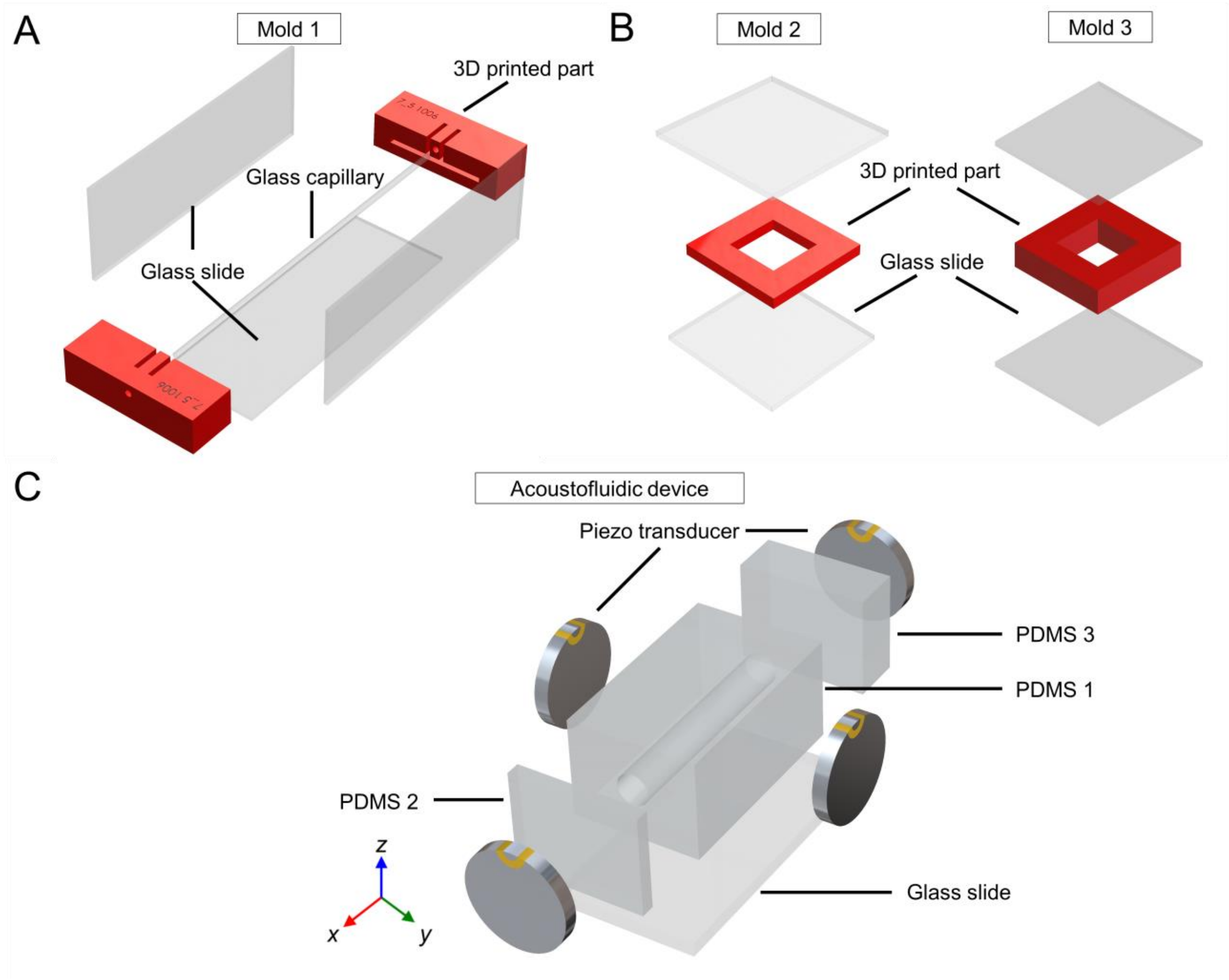

Fig. S1. Fabrication of acoustofluidic device. (A-B) Exploded view of molds for PDMS fabrication. **(C)** Exploded view of acoustofluidic device, which comprises of three transparent PDMS blocks and four identical piezo transducers.

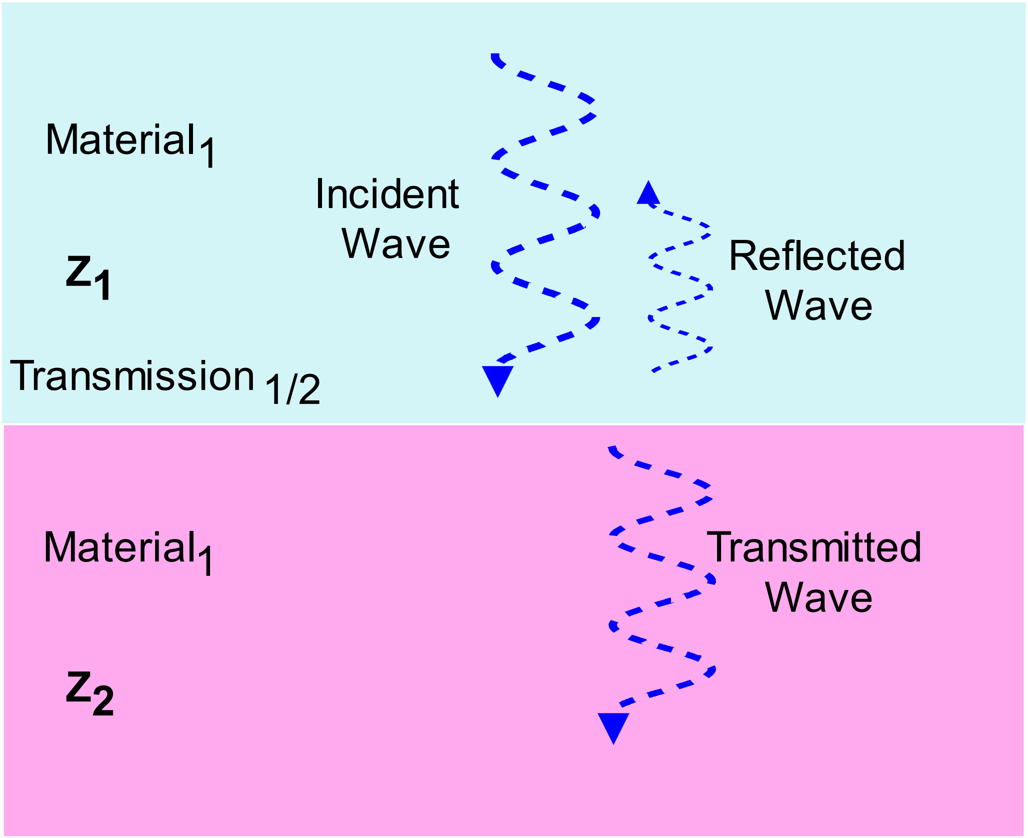

Fig. S2. Acoustic transmission and reflection at interfaces. An incident acoustic wave gets partially reflected and transmitted at an interface between a material with acoustic impedance Z_1 and a material with acoustic impedance Z_2 according to equations in Table S1.

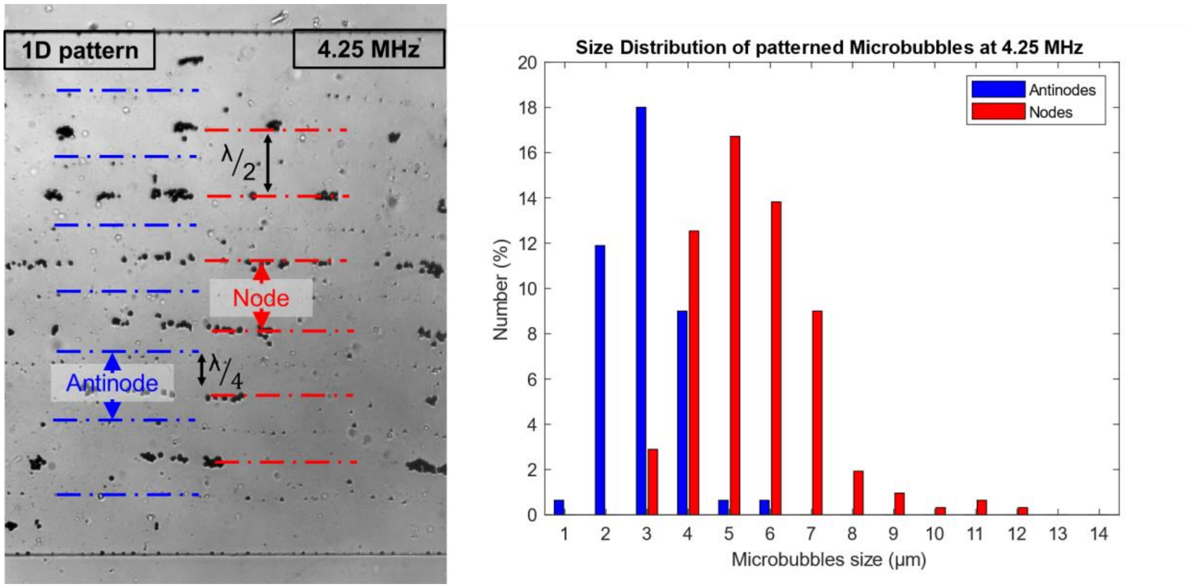

Fig. S3. Characterization and patterning of "SonoVue" microbubbles within a microchannel. (A) Image of patterned lines of microbubbles inside a one-dimensional acoustic field within a microchannel. **(B)** Plot of the size distribution of the patterned microbubbles.

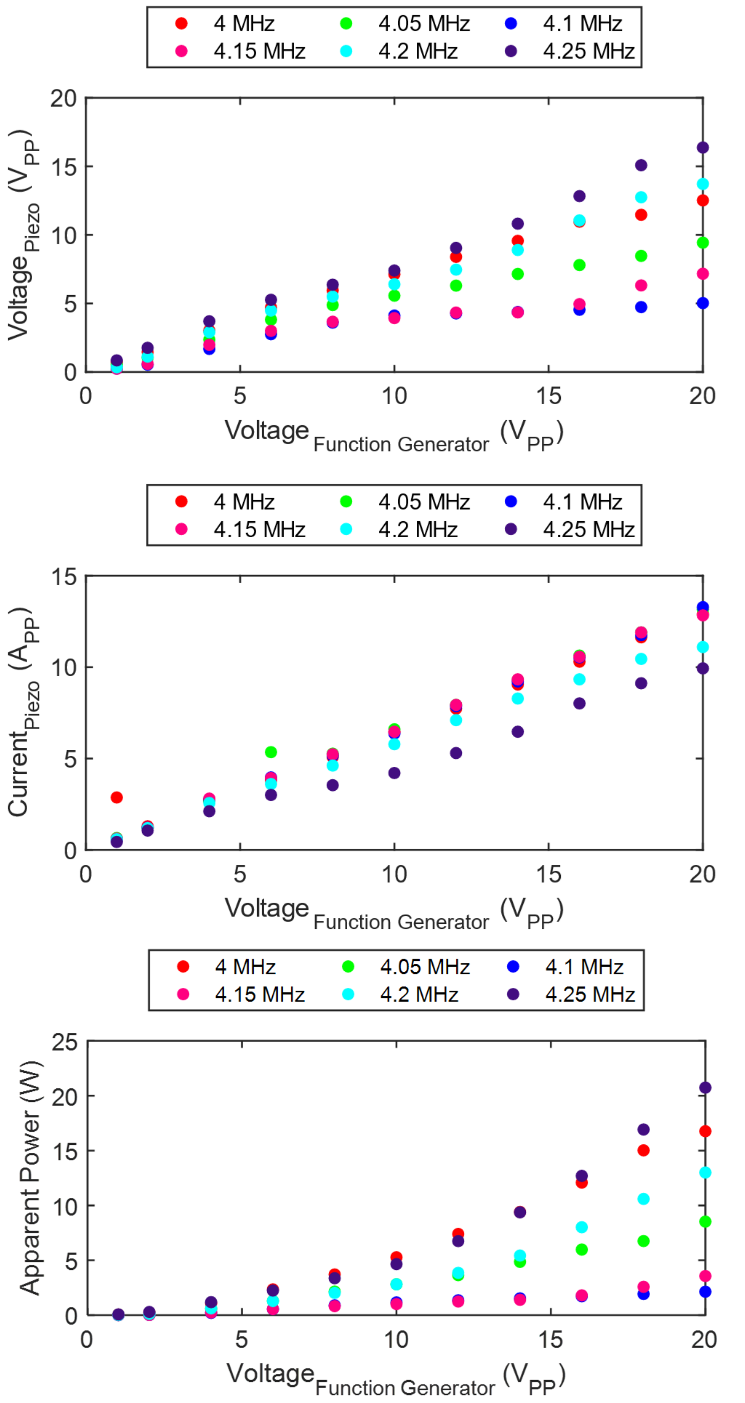

Fig. S4. Measurements of actuation signals. (A) The peak-to-peak voltage measured at the piezo transducer. **(B)** The peak-to-peak current measured at the piezo transducer. **(C)** The apparent power, which is determined by multiplying the voltage RMS and current RMS.

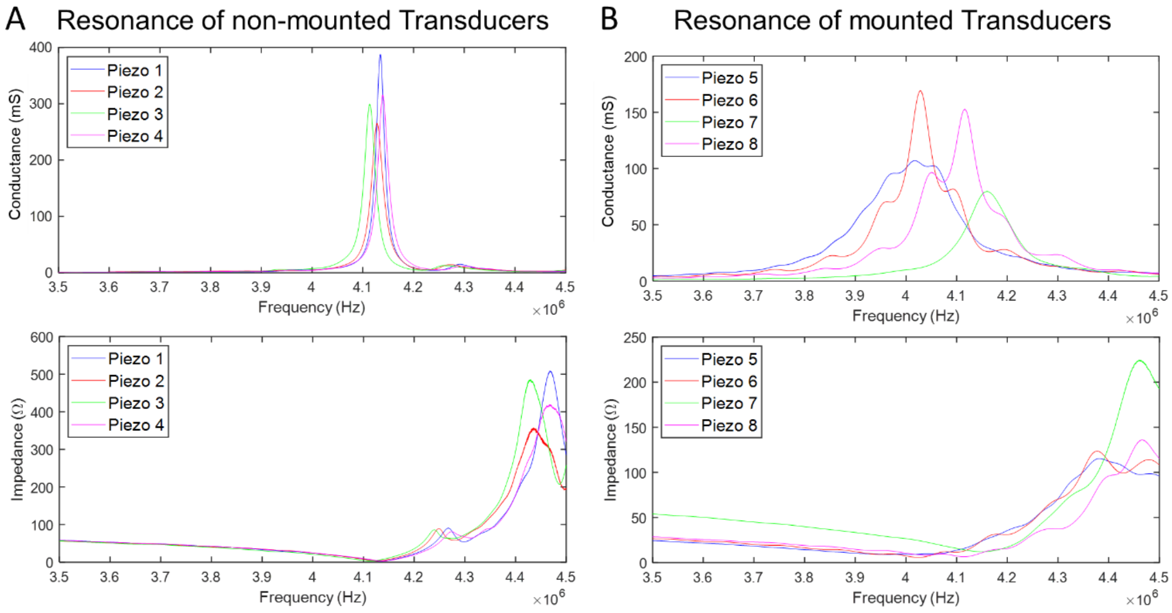

Fig. S5. Measurement of the electrical resonane of the piezo transducers. As the excitation voltage approaches its resonance frequency, the piezo transducer's conductance increases and impedance decreases. Measurement of **(A)** conductance, and **(B)** impedance of four unmounted and four mounted piezoelectric transducers.

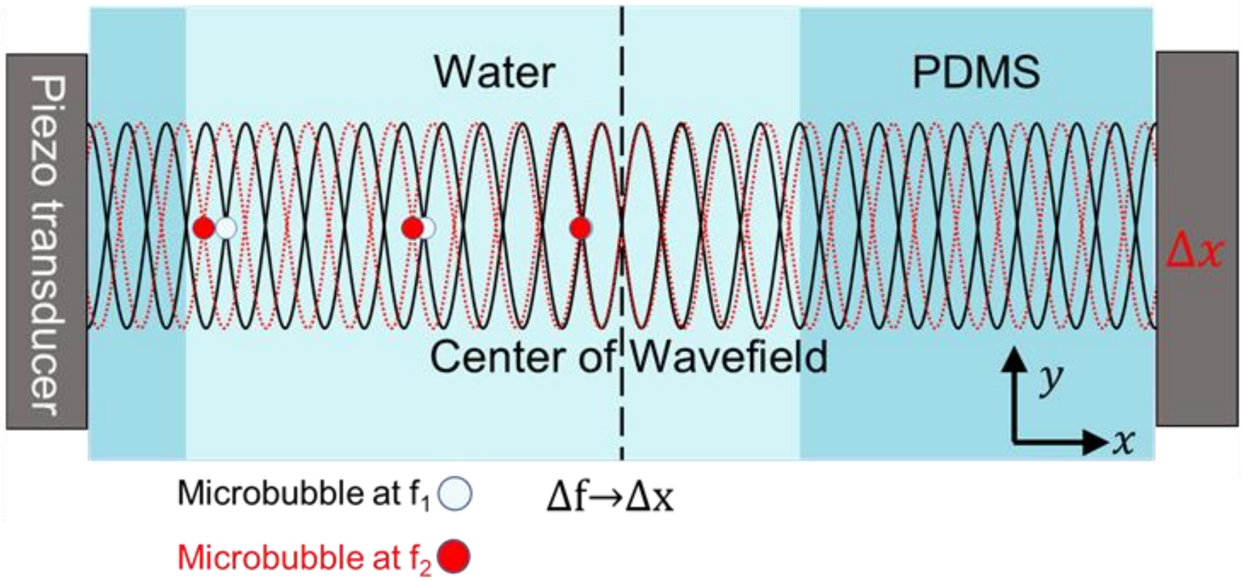

Fig. S6. Schematic of acoustically trapped particles changing their position. The particle displacement is proportional to their distance from the center of the wavefield when the acoustic frequency changes by Δf. The original position of the microparticles is indicated by the white circles. The displaced particles are indicated by the red circles.

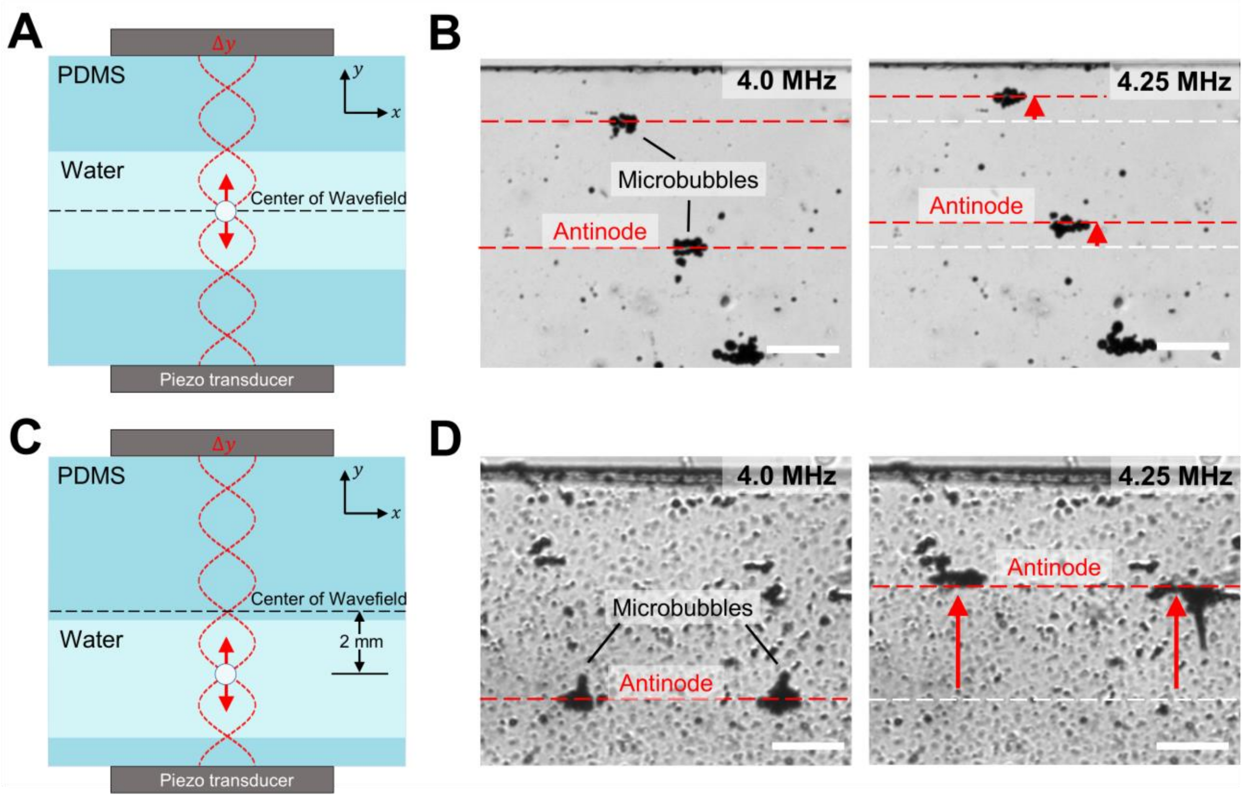

Fig. S7. Acoustic position manipulation through frequency change. (A) Schematic of an acoustic device with a centrally placed microchannel. Acoustic standing wave indicated by the red line and control direction of microbubbles indicated by red arrows. **(B)** In a centrally placed microchannel microbubbles change their position by 29 µm when the actuation frequency changes from 4 MHz to 4.25 MHz (scale bar, 100 µm). **(C)** Schematic of an acoustic device with a microchannel placed 2 mm off-center. **(D)** In a 2 mm off-center placed microchannel the same change in frequency leads to a change of 167 µm in the position of the microbubbles (scale bar, 100 µm).

Supplementary Tables

Acoustic Impedance	Acoustic Transmission Coefficient
$Z = \rho \cdot c$	$\prod_i T_i = \left[1 - \left[\frac{Z_{i+1} - Z_i}{Z_{i+1} + Z_i}\right]^2\right]$
$Z_{PDMS} = 1.05 \cdot 10^6 \, kg/(sm^2)$	$T_{PDMS/Agar} = 1 - \left[\frac{Z_{Agar} - Z_{PDMS}}{Z_{Agar} + Z_{PDMS}}\right]^2 = 96.1\%$
$Z_{Agar} = 1.57 \cdot 10^6 \, kg/(sm^2)$	$T_{Agar/Soft\ tissue} = 1 - \left[\frac{Z_{Soft\ tissue} - Z_{Agar}}{Z_{Soft\ tissue} + Z_{Agar}}\right]^2 = 99.9\%$
$Z_{Soft\ tissue} = 1.63 \cdot 10^6 \, kg/(sm^2)$	$T_{Soft\ tissue/Blood} = 1 - \left[\frac{Z_{Blood} - Z_{Soft\ tissue}}{Z_{Blood} + Z_{Soft\ tissue}}\right]^2 = 99.9\%$
$Z_{Blood} = 1.66 \cdot 10^6 \, kg/(sm^2)$	$T_{Soft\ materials} = T_{PDMS/Agar} \cdot T_{Agar/Soft\ tissue} \cdot T_{Soft\ tissue/Blood} = 96.0\%$

Table S1. Material parameters for acoustic transmission. Acoustic impedances Z_i and acoustic transmission coefficients T_i of the experimental setup [47, 50, 51].

Transducer	f_{res}	Q_{factor}
Piezo 1	4.134 MHz	320.5
Piezo 2	4.129 MHz	225.6
Piezo 3	4.114 MHz	236.5
Piezo 4	4.140 MHz	257.2
Piezo 5	4.048 MHz	26.3
Piezo 6	4.034 MHz	93.0
Piezo 7	4.173 MHz	54.7
Piezo 8	4.119 MHz	93.0

Table S2. Resonance frequency and quality factor of four unmounted and four mounted transducers during conductance and impedance measurement in fig. S5.

Supplementary Movies

Movie S1.
2D-patterning and 2D-manipulation of microparticles in a microchannel. 2×real time, 17.2 fps, scale bar 100 µm

Movie S2.
Acoustic patterning of microbubbles at 4.0 MHz in microchannel. real time, 8.6 fps, scale bar 100 µm

Movie S3.
Manipulation of microbubbles in a centred microchannel. 2×real time, 17.2 fps, scale bar 100 µm

Movie S4.
Manipulation of microbubbles in a 2 mm off-centre microchannel. 2×real time, 17.2 fps, scale bar 100 µm

Movie S5.
Microbubble trapping in dorsal aorta of zebrafish embryo. real time, 8.6 fps, scale bar 50 µm

Movie S6.
Microbubble trapping in dorsal aorta of zebrafish embryo. real time, 8.6 fps, scale bar 25 µm

Movie S7.
Microbubble manipulation upstream in post cardinal vein. real time, 8.6 fps, scale bar 25 µm

Movie S8.
Microbubble manipulation downstream in dorsal aorta and upstream in post cardinal vein. 3×real time, 25.8 fps, scale bar 25 µm

Movie S9.
Repeated microbubble manipulation crossstream in dorsal aorta. 2×real time, 17.2 fps, scale bar 10 µm

Movie S10.
Repeated microbubble manipulation up- and downstream in post cardinal vein. real time, 8.6 fps, scale bar 25 µm

Movie S11.
Repeated microbubble manipulation up- and downstream in dorsal aorta. real time, 8.6 fps, scale bar 25 µm

Movie S12.

Repeated microbubble manipulation up- and downstream in intersegmental vessel, real time, 8.6 fps, scale bar 25 µm

Movie S13.

Repeated microbubble manipulation up- and down-stream in a cerebral capillary in the ZFE head. real time, 8.6 fps, scale bar 25 µm

Movie S14.

Effects of the acoustic field (10 V_{PP}) on cardiac function in ZFE. real time, 8.6 fps, scale bar 50 µm

Movie S15.

Effects of the acoustic field (15 V_{PP}) on cardiac function in ZFE. real time, 8.6 fps, scale bar 50 µm

Movie S16.

Effects of the acoustic field (20 V_{PP}) on cardiac function in ZFE. real time with a 1 mm air gap acoustic insulator. real time, 8.6 fps, scale bar 50 µm
